# Connecting Texture and Breakup in Water and Simulated Gastric Fluid with Different Food-like Tablets

**DOI:** 10.3390/foods15081297

**Published:** 2026-04-09

**Authors:** Jingying Cheng, Timothy Langrish

**Affiliations:** Drying and Process Technology Research Group, School of Chemical and Biomolecular Engineering, The University of Sydney, Camperdown, NSW 2006, Australia; jche0791@uni.sydney.edu.au

**Keywords:** in vitro dissolution breaker system, microcrystalline cellulose, apple pectin, breakup, Young’s modulus, interfacial shear stress

## Abstract

Three food-like tablet types, with Young’s moduli similar to those of real foods, were prepared to investigate breakup during digestion using caffeine as a model solute. Texture was evaluated in situ during simulated digestion by measuring Young’s moduli and fracturability at various time points, providing indicators of stiffness and toughness. Type 1 disintegrated immediately; Type 2 dissolved first, followed by breakup at (1.5 ± 0.2) min, and Type 3 underwent dissolution. Young’s modulus decreased rapidly for Type 1 within a minute (from 1.00 to 0.38 MPa), while Type 2 exhibited a decrease at 1.5 min (0.94–0.58 MPa) before breakup. Type 3 resisted disintegration due to its higher modulus of elasticity. The time-dependent decrease in Young’s modulus is consistent with previous studies, suggesting that soft materials are more readily broken down. In simulated gastric fluid (SGF), Type 2 displayed similar dissolution and breakup behaviour (1.8 ± 0.04) min, followed by structural stabilisation due to swelling, with a slight decrease in modulus and fracturability at breakup. The study introduces a novel method that combines time-resolved, in situ textural measurements with real-time visual observation under physiologically relevant pulsatile flow, using purpose-designed food-like model materials to support the prediction of food breakdown behaviour and the design of foods with controlled digestion.

## 1. Introduction

Food disintegration begins with mastication in the mouth [1,2,3,4,5,6]. The high normal and shear stresses, together with high shear rate, cause mechanical breakdown [1], where large pieces of food are fragmented into small pieces [7]. The small pieces of food continue to be lubricated by saliva, and the food particles become a food bolus, which subsequently moves into the stomach [6,8].

The primary function of the stomach includes mixing, grinding and sieving, which is the main digestion and storage area for food [9,10,11,12]. The storage occurs in the fundus and body, whereas the grinding and mixing functions occur in the antrum [13]. One of the mechanisms of disintegration is erosion, characterised by the abrasion of particle surfaces caused by fluid shear stress [11,14], and due to hydrodynamic mixing [15]. Then the food particles are broken down into smaller particles (typically less than 1–2 mm) to pass through the pylorus [10,14,16,17,18,19].

Bornhorst et al. [20] proposed a Food Breakdown Classification System (FBCS), which used initial hardness and softening rate as the main physical properties to classify different foods and their breakup behaviours. Therefore, the initial hardness and rate of softening of the food were measured in some of the literature when analysing the breakup process [21,22,23,24]. Other papers have measured different physical properties parameters. The fracture stress and strain have been measured for developing model solid foods, which provides insight into the relationship between the mechanical properties and the food breakup during digestion [23,25].

Food texture and other aspects of structure affect how macronutrients break up and how nutrients are released during digestion, thereby affecting nutrient bioavailability [26]. The decreasing particle size increases the surface area of food that breaks up during digestion, improving digestion and nutrient absorption, which are both governed by bioaccessibility and bioavailability [26,27]. Therefore, designing food texture to control nutrient release can help improve nutritional outcomes from existing foods without altering the food composition. This approach also supports food sustainability by obtaining more nutrition from existing food resources to reduce waste.

Regarding the food structure, the Young’s moduli of coated bilayer tablets in biconvex form are around 32 MPa [28], while the Young’s moduli for foods range from 0.028 MPa for bananas to 8.8 MPa for carrots [29]. We formulated tablets with deliberately low Young’s moduli (0.4–1.6 MPa) to better simulate food breakup with some degree of realism for solid foods. The tablet range encompasses many soft foods, rather than the much higher moduli typical for pharmaceutical compacts. The creation of food-like tablets, in terms of their Young’s moduli, using spray-dried powders is a novel feature of this work and allows these tablets to be used, which have food-like stiffness and hardness.

Pectin can gel in the presence of acid and calcium ions, especially low-methoxyl (LM) pectin [30]. The gelation of the pectin can induce swelling pressure, interfacial stress and confinement stress. As the pectin network forms and absorbs water, ionic crosslinking induces swelling pressure and causes expansion, which helps exert internal stress (axial and radial stress) within the tablets [31]. Microcrystalline cellulose (MCC) is generally inert and provides mechanical strength and compressibility; however, it may resist swelling [32], increasing internal stress during the loss of caffeine due to mass transfer.

In this study, the newly developed dissolution beaker system was introduced to simulate pulsatile flow conditions and was combined with time-resolved, in situ texture analysis to quantitatively relate texture (Young’s moduli and fracturability) to the breakup behaviour of food-like tablets in water and SGF under pulsatile flow. This framework helps to improve understanding of the interaction between changes in food texture and breakup during the digestion process. In all the tablets, caffeine was used as the model solute, since caffeine is chemically stable, well-characterised, and widely occurring in food and beverages, such as coffee, tea and cocoa-based products and energy drinks [33], making caffeine relevant and practical as a model compound. Furthermore, Cheng et al. [34] studied the slow-release behaviour of tablets by using caffeine, which informed the design of tablets in this study.

## 2. Materials and Methods

### 2.1. Experimental Chemicals

Pectin (apple) powder was purchased from Morlife Pty Ltd. (Arundel, QLD, Australia), while caffeine powder (1,3,7-trimethylxanthine, laboratory grade) was purchased from Merck Life Science Pty Ltd. (Melbourne, VIC, Australia) and the microcrystalline cellulose powder (90 microns) were purchased from Clentham Life Sciences (Corsham, UK). Concentrated hydrochloric acid (32%) (HCl) (RCI Labscan, Bangkok, Thailand), sodium chloride (NaCl, laboratory grade), and pepsin (1:2500, laboratory grade) were obtained from Chem-Supply Australia (Gillman, SA, Australia).

### 2.2. Experimental Equipment and Methods

In this study, a newly developed beaker dissolution system was designed to characterise pulsatile flow conditions, and a simplified texture analyser was developed to enable time-resolved, in situ measurements. The combination of new equipment and the use of food-like textures enables quantitative analysis of the relationship between breakup behaviour and texture.

#### 2.2.1. Powder Preparation

##### Spray Drying

The Buchi Mini Spray Dryer B-290 (Flavil, Switzerland) was used to dehydrate the previously prepared aqueous mixture of pectin and caffeine [34] and the homogeneous suspension of caffeine and microcrystalline cellulose, prepared at specific ratios. A solution with 15 g/L (90%) apple pectin and 1.67 g/L (10%) caffeine was prepared in 300 mL of deionised water (DI water) under stirring at room temperature. The solution of microcrystalline cellulose (MCC) and caffeine was prepared using the same process, with 15 g/L (90%) MCC and 1.67 g/L (10%) caffeine, and caffeine was dissolved in the DI water at room temperature, then MCC was gradually added and dispersed under continuous stirring to obtain a homogeneous suspension. Then the Buchi-B290 Mini Spray Dryer was set up to spray dry the feed solution. The operating conditions, as described by Cheng et al. [34], consisted of a liquid flow rate of 7.5 mL/min and an inlet air temperature of 190 °C, with a main air flow rate of 35 m^3^/h.

##### Physical Mixing

A Vortex mixer (Ratek, Melbourne, Australia) was also used to help with the physical mixing of the microcrystalline cellulose and caffeine. A mass of 0.18 g of MCC (90%) and 0.02 g (10%) of caffeine was weighed out, and then all the powders were put into a tube. The tube was placed on a Vortex mixer for 15 s to mix the powders physically before making the tablet.

#### 2.2.2. Tablet Design

##### Tablet Composition

Tablets were used as model foods because their composition may be relatively easily controlled, enabling their breakup and dissolution behaviour to be determined with some degree of reproducibility. MCC is extensively employed as a disintegrating agent in both dry compression and wet granulation methodologies [35]. MCC was therefore considered one of the components of tablet formulation. The composition of the different tablets (Table 1) was formulated either from physically mixed or spray-dried powders. Spray-drying technology was used as a powder preparation method, which is effective way of mixing and producing homogeneous powders from liquid feeds [34,36].

Cheng et al. [34] studied internal and external mass-transfer resistances governing caffeine release from pectin, in which tablets with a 9:1 ratio of caffeine and pectin were formulated as fast-release tablets. Their study showed low internal resistance to caffeine transport through pectin for the 90% caffeine mixture. The effect of excipient ratio on stress dispersion has also been demonstrated in mixed tablets of porous calcium silicate (FLR) and phenacetin (PHE), where small FLR additions (9:1) increased the stress concentrations and the dissolution rates of PHE [37]. This previous work using a caffeine:pectin ratio (9:1) motivated the selection of this ratio in this study.

A Logitech HD Pro Webcam C920 camera was used to record the behaviour of the tablets in the solutions. The transparency of the new beaker system enables it to be used to directly observe the tablet breakup and particle release.

##### Tablet Making

A hydraulic press machine manufactured by TMAX (Xiamen, China) was used to compress the tablets. A 0.2 g sample of the spray-dried pectin–caffeine or MCC–caffeine powder was weighed and then placed into a tablet maker. Additionally, the physically mixed powder (0.2 g) was blended for 15 s before compression into a tablet. All samples were then subjected to the same tablet compression process (1 tonne, 30 s), with a compressive stress of 74 MPa.

##### Tablet Design for Texture

The Young’s moduli of the tablets, measured at different digestion time points and ranging from 0.4 to 1.6 MPa, were compared with values reported for existing food materials (Table 2). These comparisons with existing food materials showed that the tablets here were within a representative range for the Young’s moduli of soft foods, such as cheese and cooked vegetables, supporting the food-like characteristics of our tablets, at least in terms of their initial hardness.

Food disintegration can be affected by moisture uptake, which reduces brittleness [43]. Bornhorst and Singh [6] reported that the fracture stress and Young’s modulus are related to the mechanisms of breakdown, with the fracture stress representing material toughness. The limitation of this study is that it mainly measured Young’s modulus, simplifying the representation of other physical properties involved in the breakup process during digestion. However, this study analyses the initial breakup under pulsatile flow conditions. Using Young’s modulus as a criterion provides a practical and controlled, but simplified, method for understanding the first steps in the breakup process [43].

#### 2.2.3. Dissolution Test System

**Figure 1:**
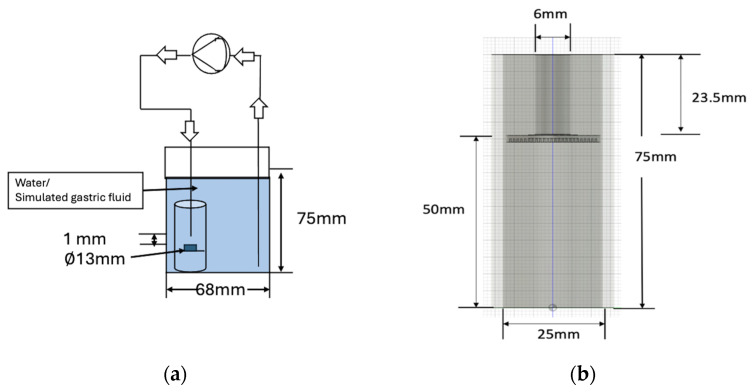
(**a**) Front view showing tablet position in the 3D-printed holder within system. (**b**) Dimensions of the pulsatile flow tube and holder assembly.

A newly developed beaker system was used to simulate the gastric environment. The system was similar to a USP (U.S. Pharmacopeia) Type 3 apparatus, in which the flow around the tablets was a transient, reciprocating process rather than smooth and steady [44]. This transient process was created by a diaphragm pump (Hanna Instruments, Woonsocket, RI, Australia). A 3D-printed holder (Jaycar Electronics, Sydney, Australia) was used to position the tablet within the pulsatile flow field (Figure 1), in which the distance of the tablets from the bottom of the holder was 50 mm. A tube was put into the beaker system to help maintain the position of the tablet holder. The tablets were placed on the holder. The pump flow rate was set to the maximum flow rate, which corresponded to a net (average) flow rate of 10.8 L/h, and the frequency of the diaphragm pump was set to 120 strokes per minute. The diameter of the tube for the diaphragm pump was 6 mm (Figure 1b). The diameter of the tablet was 13 mm, as shown in Figure 1a. A 250 mL beaker containing 230 mL of solution (deionised (DI) water or simulated gastric fluid) was used in this experiment. The fluid flows in the new beaker system were characterised by a transient pulsatile pattern from the pump, whereas those in the old beaker system were steady [34].

Simulated gastric fluid (SGF) was prepared by dissolving 3.2 g of pepsin and 2.0 g of sodium chloride in Milli-Q water (Merck, Darmstadt, Germany). Subsequently, 7.0 mL of concentrated hydrochloric acid (HCl) was added, and the solution was adjusted to a final volume of 1.0 L with Milli-Q water. The final pH of the solution was about 1.7 [34,45,46].

**Figure 2:**
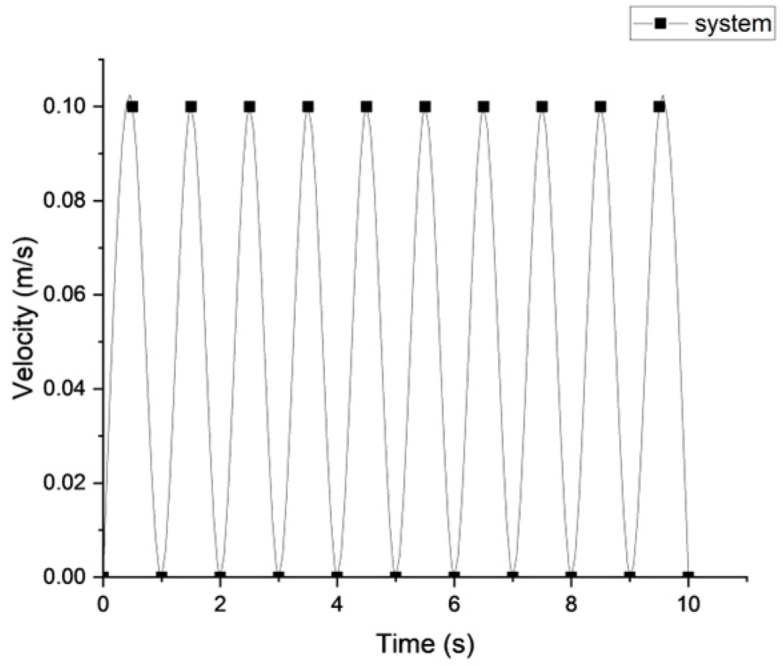
Velocity–time curve at the outlet of the pump pipe in the new beaker system, upstream of the tablet.

The velocity–time behaviour of the new beaker system (Figure 2) was calculated based on measurements obtained from the pump outlet when operated at 120 strokes/minute. The upper stomach wall normally contracts at a frequency of about 3 cycles/minute [47]. The operated pump frequency (120 strokes/minute) provided an accelerated-time digestion process, whereby one minute of experimental test represents 40 min of real digestion, while maintaining the peak velocity to be consistent with the reported velocity value. The TIMagc model was developed to simulate the migrating motor complex (MMC) phases I and II in the fasting state, with maximum velocities of 0.19 m/s in phase I and 0.081 m/s in phase II [48]. Kuhar et al. [49] found that the maximum velocity in the pylorus of a healthy stomach was 0.1 m/s, with the fluid viscosity of 1 × 10^−3^ Pa s. Therefore, the maximum velocity in our system of 0.1 m/s (Figure 2) falls within the range of the simulated velocities generated in the stomach by antral contractions. The maximum velocity was calculated from the maximum flow rate of the outlet pipe, which was positioned upstream of the tablet.

#### 2.2.4. Texture Analyser

**Figure 3:**
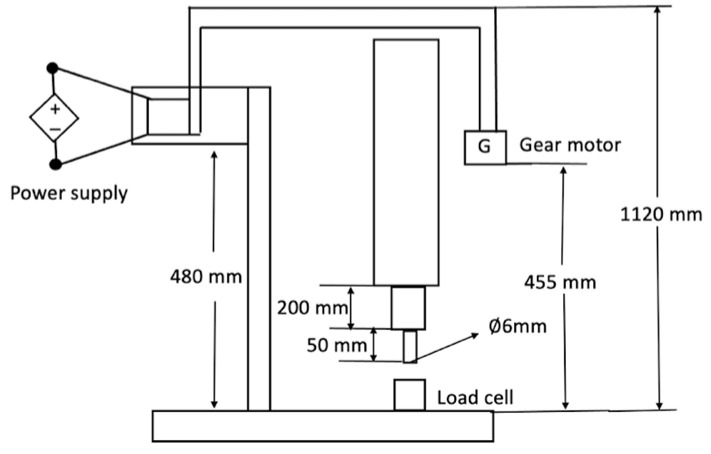
Schematic diagram of texture analyser.

A texture analyser was developed for the experiment with a load cell (Siemens, Munich, Germany), a DC power supply (Powertech, Malaga, Western Australia) connected to a gear motor (MFA/Como Drills, Bangor, UK), which controlled the transient shear strain rate (Figure 3). The optional voltage range was 4.5 to 15 V, and the maximum power drawn from the power supply was 12 W. The sample was placed on the load cell, and the gear motor drove the probe through the sample at a constant velocity, controlled by the applied direct current voltage. The crosshead speed was 0.5 mm/s. The load cell (Siemens, Munich, Germany) was connected to the computer via a Picolog ADC-24 data acquisition system (Picolog Technology, St Neots, UK), which recorded the voltage output from the load cell, with a capacity of up to 5 kg. The output sample interval was set at 60 milliseconds.

When the probe contacted the sample, the system recorded changes in apparent mass (force = mass × g, where g is the acceleration due to gravity). The probe was a cylindrical wooden stick with a diameter of 6 mm to apply localised compression. A puncture test protocol with a 6 mm diameter probe was developed by Christofi et al. [50] for testing canned peach halves. The puncture test has also been used to evaluate the internal structure of the tested sample [50,51]. Therefore, a 6 mm cylindrical probe was considered appropriate for investigating the internal structure of the tablets.

Subsequently, voltage–time data were exported as .csv files for analysis using Microsoft Excel. The voltage and mass calibration curve was measured using a load cell and standard calibration masses. When texture was measured, the tablet was initially placed into the new beaker system at specific time points (0.5 min, 1 min, 1.5 min, 2 min, 2.5 min, and 3 min). At each designated time point, the tablet was removed from the new beaker system and positioned on the load cell to determine its textural properties, specifically the Young’s modulus or modulus of elasticity. The texture analyser was positioned close to the breakup dissolution system to minimise the moisture loss when removing samples from the beaker system and then replacing them in the beaker system. The time between removal from the fluid, the transfer time, was under 30 s for all samples. Although this procedure involves some destructive testing and may result in slight changes to surface moisture, it was applied to all samples and repeated three times to ensure repeatability.

**Figure A2:**
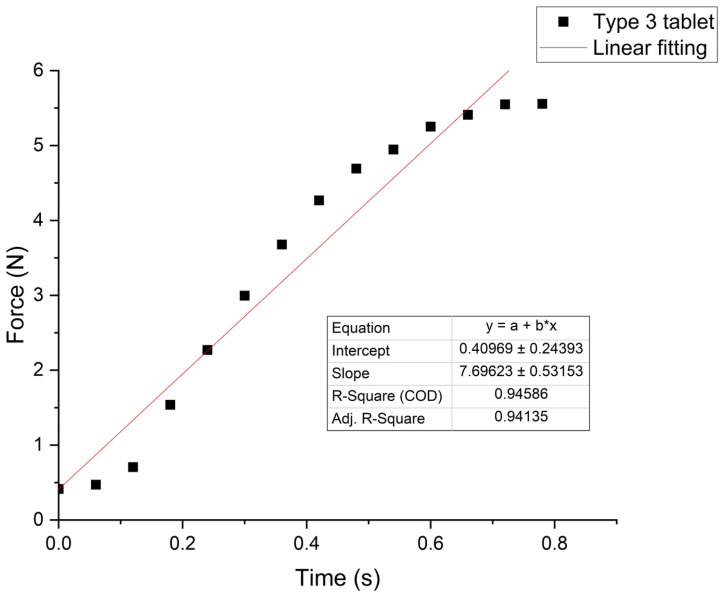
Typical force–time curve used to calculate the Young’s modulus (about 95% confidence).

Young’s modulus is defined by the following equation [52]:

(1) $$E=\frac{F}{∆t}÷\frac{∆L}{∆t}\times \frac{L_{0}}{A}$$

where *F/*Δ*t* is the force rate, where the slope of the linear elastic region of the force–time curve is shown in Figure A2, *L*_0_ is the height of the sample, *A* is the cross-sectional area, and Δ*L/*Δ*t* is the length change with respect to time. The compressive force acts on the sample surface in a perpendicular direction, which is defined as a normal stress [53]. Therefore, Young’s modulus is related to the normal stress in this situation.

**Figure 4:**
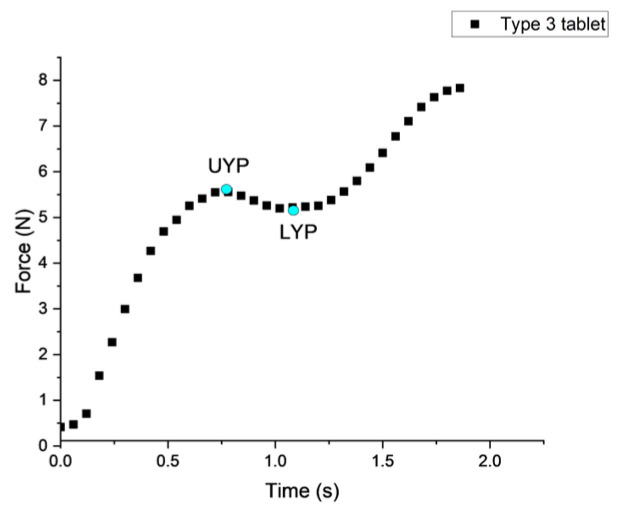
Schematic diagram of a typical texture analysis test for a Type 3 tablet. UYP and LYP represent the energy corresponding to the upper and lower yield points, respectively.

Fracturability was defined as the upper yield (UYP) point, which is the first major drop in the force–time curve, while LYP means the lower yield point, as shown in Figure 4 [52,54].

#### 2.2.5. Microcomputed Tomography (Micro-CT)

Microcomputed tomography (micro-CT) was used to study the internal structure of the tablets (Bruker, Preston, VIC, Australia). Micro-CT analysis was performed at the Australian Centre for Microscopy and Microanalysis, University of Sydney, Australia. Two tablet samples were analysed: one obtained from the water system after one minute and the other from the SGF. The specimens were scanned using a SkyScan2214 system with a voltage of 50 kV and a current of 200 μA, with a 0.25 mm aluminum filter to reduce beam hardening. The pixel size of the image was about 14 μm, and the exposure time was 762 ms.

## 3. Results and Discussion

### 3.1. Disintegration Behaviour and Texture Development

#### 3.1.1. Tablets in Water

**Figure 5:**
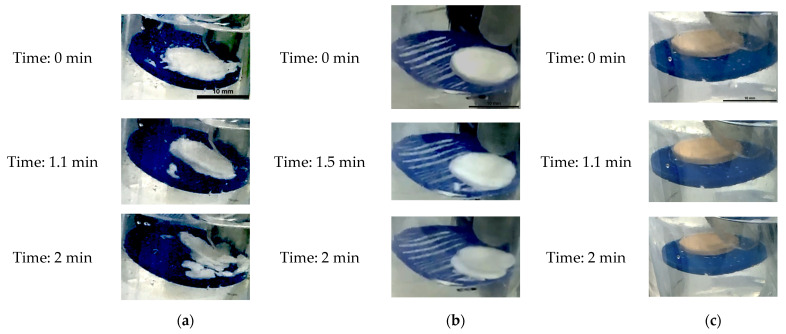
Typical images obtained in water for different tablet dissolution and breakup in the new beaker system at various times: (**a**) Type 1 tablet, (**b**) Type 2 tablet, (**c**) Type 3 tablet.

The Type 1 tablet condition was recorded at t = 0 min, as shown in the first image, and the tablet showed a breakup process (Figure 5a). The Type 2 tablet showed a different breakup process compared with the Type 1 tablet. The breakup of the Type 2 tablets cooccurred at (1.5 ± 0.2) minutes. For Type 3 tablets, dissolution occurred without a visible change in size between 0 and 2 min (Figure 5c). Therefore, when comparing the two spray-dried formulations, the pectin–caffeine tablets (90% pectin + 10% caffeine, Type 3) followed a slow dissolution process, whereas the MCC–caffeine tablets (90% MCC + 10% caffeine, Type 2) initially followed a rapid dissolution process. MCC is employed as a disintegrating agent [35]. Therefore, tablets containing microcrystalline cellulose (Types 1 and 2) showed disintegration behaviour in water.

#### 3.1.2. Type 2 Tablets in SGF

The Type 2 tablet showed a more stable disintegration process compared with the Type 1 tablet. Simulated gastric fluid was used to mimic the physiological conditions of the stomach. As a result, the Type 2 tablet was considered more suitable for studying the breakup process under simulated gastric conditions, which better reflected the in vivo gastric environment.

**Figure 6:**
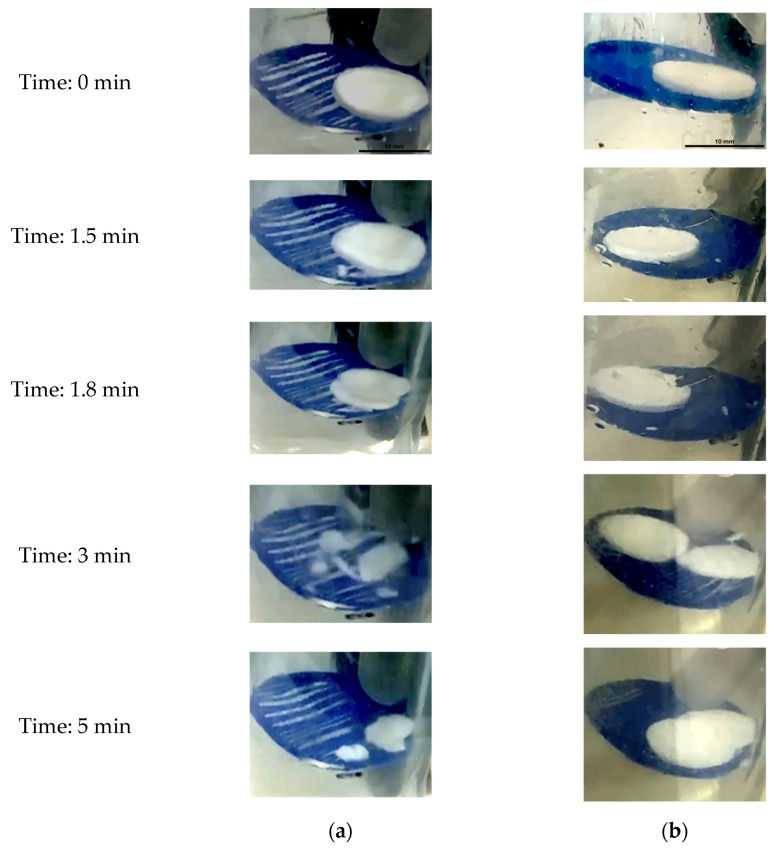
Typical images obtained during the new beaker system at various times: (**a**) Type 2 tablet in water, (**b**) Type 2 tablet in SGF.

The behaviour of the Type 2 tablet in water is shown in Figure 6a, while its behaviour in simulated gastric fluid (SGF) is shown in Figure 6b. A breakup phenomenon occurred at (1.8 ± 0.04) minutes in the SGF system (Figure 6b), followed by structural stabilisation at (3 ± 0.09) minutes. Following the breakup, the tablet largely retained its shape at the point of fracture. In comparison, the Type 2 tablet in water exhibited a more rapid breakup process than the tablet in SGF.

MCC is typically prepared through acid hydrolysis of cellulose, a method that removes amorphous regions and increases the crystallinity index of the material [55]. The stability of the crystalline regions of MCC is affected by their resistance to acid degradation [56,57]. The increase in moisture absorption of the tablet can be observed when the crystallinity of MCC is decreased [58]. MCC is a disintegrant that promotes tablet breakup by absorbing water, leading to swelling and mechanical rupture [35]. Therefore, the Type 2 tablet was observed to maintain a stable structure after three minutes in an acidic solution, while it broke up in water.

Each test was repeated three times, resulting in a mean breakup time for Type 2 tablets of (1.5 ± 0.2) minutes and (1.8 ± 0.04) minutes in SGF. These breakup times are quite similar, suggesting that any differences between the Type 2 tablets in water and in SGF are initially small. The Type 1 tablet underwent immediate breakup, with a breakup time of (0.08 ± 0.004) minutes.

#### 3.1.3. Discussion of the Fluid Shear Stress and Breakup Mechanisms

Nakata et al. [59] calculated the wall shear stress for pulsatile flow using $\tau =\mu \frac{du}{dy}$, where *y* was 0.5 mm, which is the distance from the wall at which velocity was measured. In this study, the velocity profile was measured at the pump tube point, and the distance from the pump tube to the tablet surface was 1 mm, as shown in Figure 1a. The maximum velocity was 0.1 m/s, as shown in Figure 2.

In this system, the maximum wall shear stress on the tablet surface may be estimated as τ = 0.001 Pa·s × 0.1 m/s ÷ 0.0001 m = 0.1 Pa. These shear stresses are several orders of magnitude less than the measured strengths of the tablets, which are all above 0.2 MPa. In the computer simulation, the range of shear stress in vivo in the stomach has been reported to be about 10–70 dyne/cm^2^ (1–7 Pa) [60]. Therefore, the fluid shear stress in the system, compared with the tablet strength, suggests that the fluid shear stress may not be the primary reason causing disintegration. These results indicate that fluid shear stress is likely to be negligible and that internal stresses within the tablets, generated by acid hydrolysis and pectin gelation, are the dominant contributors to tablet breakup.

With respect to other digestion devices, TIMagc was developed based on the TNO model, which estimated the shear rate to be between 0.001 and 360 s^−1^ with a viscosity of 20 cP, resulting in a shear stress range of 0.6–7.2 Pa [48]. The human gastric flow simulator (GFS), which incorporates peristaltic motion, generates shear stresses that range from 0.01 to 3 Pa [12,61]. Both systems aim to mimic the mechanical forces in the stomach during digestion. The estimated fluid shear stress in our system (0.1 Pa) is lower than the forces simulated by TIMagc and GFS. This study was designed to provide controlled, well-mixed pulsatile flow conditions for analysing the relationship between the food texture and breakdown, with emphasis on the disintegration mechanism of erosion rather than shear-induced breakup.

### 3.2. Interactions Between the Texture and the Breakup Phenomena

#### 3.2.1. The Different Tablets in the Water System

**Figure 7:**
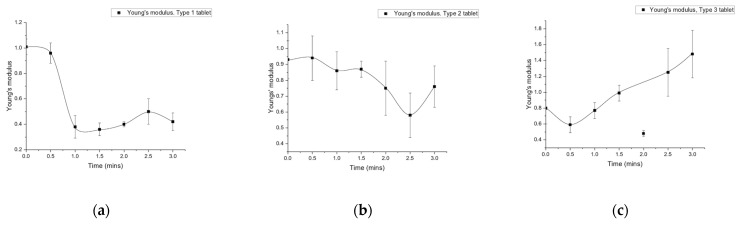
(**a**) Young’s moduli of the Type 1 tablet at different time points, (**b**) Young’s moduli of the Type 2 tablet at different time points, (**c**) Young’s moduli of the Type 3 tablet at different time points. Error bars represent the standard deviation of three repeats (n = 3).

The trend in Young’s modulus for Type 1 tablets showed a sudden decrease from 1 MPa to 0.38 MPa at one minute after the start of the dissolution (Figure 7a). After the sudden decrease, the Young’s modulus remained stable at around 0.4 MPa (Figure 7a).

Both Types 2 and 3 tablets were prepared using spray-dried powder. The Type 2 tablet showed a dissolution process from 0 to 1.5 min, while the Type 3 tablet exhibited dissolution throughout the entire observation period. For the dissolution process of the Type 2 tablet, the value of Young’s modulus decreased slowly from 0.94 MPa to 0.86 MPa (Figure 7b). The breakup occurred after 1.5 min, and the value of Young’s modulus showed a sudden decrease to 0.58 MPa (Figure 7b).

The Young’s moduli for all three types of tablets ranged from 0.48 MPa to 1.6 MPa, while in the literature, the Young’s modulus of rectangular tablets or compacts consisting of different ratios of microcrystalline cellulose (MCC) and dibasic calcium phosphate anhydrate (DCPA) ranged from 5.1 to 378 GPa [62]. The Young’s moduli for the different grades of MCC tablets ranged from 200 to 1100 MPa, while higher values from 500 to 2700 MPa have been measured for calcium phosphate tablets from 500 MPa to 2700 MPa under different compaction pressures [63]. The tablets here were intended to reflect the mechanical properties of foods. As described earlier, foods have much lower values of Young’s moduli (0.076–8.8 MPa for soft foods, 82–253 MPa for harder foods), so it was necessary to create tablets here with these lower moduli. These lower moduli were achieved by spray-drying technology, as this process produced powders with porous structures [64], where increased porosity is correlated with reduced Young’s moduli [65], showing that spray-drying methods can be used to formulate tablets with low values of Young’s moduli [64]. Plastic deformation of MCC during direct compression also helps to increase the porosity of the tablets [64,66]. Therefore, the Young’s moduli for these specially formulated tablets (0.4–1.6 MPa) were considered reasonable as model foods for analysing breakup behaviour in the digestive system.

The value of Young’s modulus for Type 1 tablets did not show a significant change from 1 min to 2.5 min (Figure 7a). However, the Type 3 tablet demonstrated an increasing trend, except at 2 min, where the value was 0.48 MPa (Figure 7c). The lower value in Young’s modulus (0.48 MPa) at 2 min may be due to the structural transition during the early stage of dissolution in water. The Type 3 tablets, composed of pectin, showed a transition from hydration-induced softening to gelling. As the gel-like structure stabilised, the Young’s modulus increased after 2 min. This trend indicated the interaction between texture and breakup phenomena, suggesting that the strength of the tablets affects their breakup, as would be expected. The texture change may be affected by time points, tablet types, and the interactions between these factors. The results of a two-way ANOVA, which are provided in Table A1, showed that the *p*-values for all three factors were below 0.05, reflecting significant differences for time points, tablet types, and their interactions.

#### 3.2.2. The Texture Variation of Type 2 Tablets in SGF

**Figure 8:**
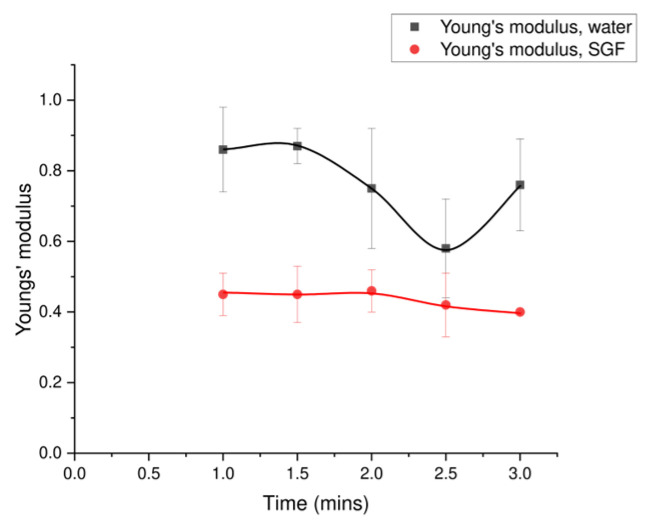
Young’s modulus of spray-dried MCC + caffeine (Type 2 tablets), which compares the tablets in different solutions. Error bars represent the standard deviation of three repeats (n = 3).

The Young’s modulus for the Type 2 tablet in water was 0.84 MPa, while in SGF it was 0.45 MPa. The trend for Young’s modulus of the tablet in SGF remained relatively stable as a function of time compared with the tablet in water. An ANOVA was performed to evaluate the effect of different time points on the Young’s moduli of the Type 2 tablet, with the detailed results shown in Table A2. A *p*-value of 0.1 was found for the effect of time, which indicated no significant difference across different time points for the Type 2 tablets in either water or SGF. A slight decrease to 0.4 MPa occurred at breakup (2 min), consistent with the observed breakup behaviour (Figure 8). This decrease corresponds to a disintegration time of 1.8 min for the tablet in SGF. The most significant effect was that of the different solutions, with supporting data provided in Table A2 (*p*-value of 4 × 10^−8^). The Type 2 tablet in water showed a sudden decrease in Young’s modulus at 1.5 min, corresponding to the breakup that occurred (Figure 8).

**Figure 9:**
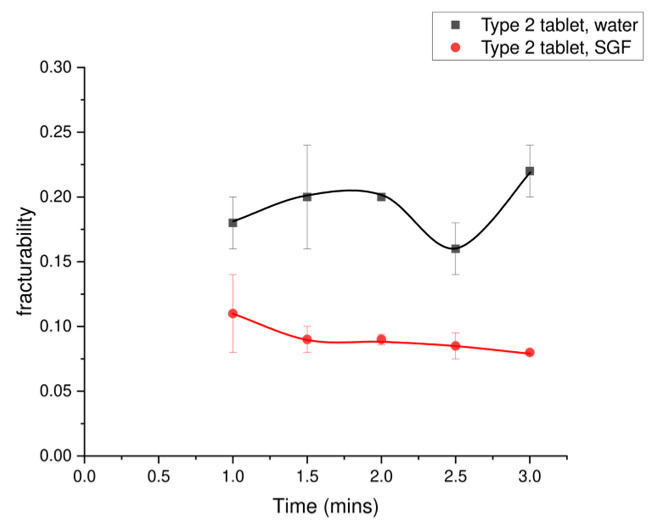
Fracturability of spray-dried MCC + caffeine (Type 2 tablets), which compares the tablets in different solutions. Error bars represent the standard deviation of three repeats (n = 3).

The value of fracturability for Type 2 tablets in SGF decreased slowly from 0.11 MPa to 0.08 MPa (Figure 9). The breakup point in SGF at 1.5 min was similar to that observed for the tablet in water (two minutes). Similarly, the Type 2 tablets in SGF and water showed the same trend between 1.5 and 2 min. After 2 min, a decreasing trend was observed, with the tablet in water decreasing five times more than the tablet in SGF. When comparing the Type 2 tablets in different solutions, the fracturability trend in SGF remained more consistent than that observed in water. The reasons for these differences in behaviour have been discussed in Section 3.1.2.

### 3.3. Microstructural Analysis for Type 2 Tablets

#### X-Ray Micro-CT Imaging

**Figure 10:**
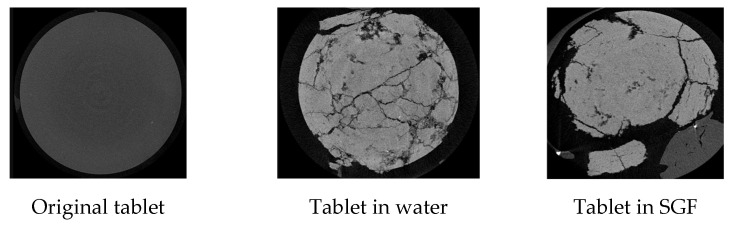
The micro-CT images of the original tablet, tablet in aqueous or SGF system for one minute.

The microstructure of the tablets in different solution systems was determined by micro-CT, which helped to explain the observed behaviour of Type 2 tablets in the water and SGF systems. The 2D micro-CT images of an original Type 2 tablet, a Type 2 tablet in water after one minute, and a corresponding tablet in SGF for one minute are shown in Figure 10. The Type 2 tablet in SGF showed fewer cracks compared with the tablet in the water system, which supported the observation that the Type 2 tablet in the SGF system disintegrated more slowly (Figure 10).

### 3.4. Overall Discussion

The food-like tablets developed in this study exhibited Young’s moduli within the range reported for real foods. Types 1 and 2 tablets, which consisted of microcrystalline cellulose (MCC) and caffeine, differed in their powder preparation methods. The Type 3 tablet contained pectin and caffeine (Table 1).

The observed difference in the behaviour of Type 2 tablets in water compared with simulated gastric fluid (SGF) was significant. A stabilised structure was observed following tablet breakup when Type 2 tablets were in SGF. Therefore, the texture change may also affect the breakup. Sun et al. [21] studied the initial Young’s modulus for different particle sizes of crackers and puree, but the texture change for different digestion times was not measured. In this study, the texture for different digestion times was measured. The initial value of Young’s modulus for the three types of tablets was about 0.9 MPa. The trends in Young’s modulus differed by type in water (Figure 7) and solution (Type 2 tablets in water or SGF), as shown in Figure 8 and Figure 9. The Type 1 tablet initially experienced a breakup process, whereas the Type 3 tablet dissolved as a complete tablet. Another study analysed Young’s modulus and texture in relation to the sensitivity of moisture content under deep-frying treatment [67]. The study showed that Young’s modulus increased with time during the deep-frying process, where an initially soft texture was observed in the potato samples [67]. Therefore, with the increased softness, an increasing trend in Young’s modulus was shown. In this work, the trend of Young’s modulus was also different between the two tablet types, where the trend for the Type 1 tablet decreased with greater times, while the Type 3 tablet showed an increasing trend. This trend may be related to the observed behaviour, where the Type 1 tablet broke up at the beginning of the process, while the Type 3 tablet (containing pectin) followed a gelation process. For the largest particle size, more rapid disintegration was observed in Kinugoshi-Tofu compared with Momen-Tofu, which may be related to differences in their mechanical properties, as the Young’s modulus of Momen-Tofu was measured to be higher than that of Kinugoshi-Tofu [68]. The Young’s modulus of the Type 2 tablet in water was found to be lower than that of the Type 3 tablet, which may be related to the disintegration observed in the Type 2 tablet.

The transformation in the behaviour of the Type 2 tablet in SGF from breakup to a stabilised structure is due to the crystallinity of MCC and its resistance to acid penetration [56,57]. The water uptake ability of microcrystalline cellulose (MCC) has been found to change over time, with microcrystalline cellulose II (MCC II) absorbing water slowly after about 15 s in simulated gastric fluid [69]. Rojas et al. [69] reported that the MCC II is produced by soaking the MCC in sodium hydroxide solution, which results in lower crystallinity and a reduced degree of polymerisation for MCC II compared with MCC. In the experiments in this work, this phenomenon was observed for the Type 2 tablet in SGF, showing the breakup followed by a stabilised structure, which was explained by the acid resistance and lower water uptake capacity.

The tablets in water showed an internal crack distribution, whereas surface-level disintegration was directly observed in the tablet in SGF in the micro-CT results. Although the breakup behaviour was observed in both media, the breakup in water is related to cracking in the internal structure, which supports further breakup in the following process, while the breakup of the tablet in SGF is dominated by radial shear stress failure caused by rapid caffeine dissolution, where the development of internal cracks is limited.

The acidic treatment breaks up the hydrogen-bonding network inside the MCC matrix, decreases the crystallinity of MCC, and increases the extent of amorphous regions [70]. The amorphous regions deform plastically, which requires less energy to fracture than crystalline regions [71]. Therefore, the value of Young’s modulus for the SGF tablet at 1 min was 0.45 MPa, which was lower (*p* = 0.05) than that of the tablet in water (0.84 MPa).

Although MCC acts as a disintegrant that promotes breakup by absorbing water, leading to mechanical rupture [35], the acid penetration resistance in SGF maintained a stabilised tablet structure. As a result, the breakup of the tablet in SGF occurred at the surface layer, while the limited internal structure cracking remains mechanically stable. The micro-CT results aligned with the trend of the stiffness (Young’s modulus) measurements, showing that the tablet in water showed more internal cracking compared with the tablet in SGF (Figure 10). The microstructure results from micro-CT further supported the observations that the Type 2 tablet in SGF had a slower water absorption, leading to slow swelling and delayed disintegration.

### 3.5. Comparative Analysis of Stress Mechanisms Behind the Different Behaviours of Various Tablet Types

#### 3.5.1. Discussion of Different Internal Behaviours for Various Types of Tablets

Wagner-Hattler et al. [72] found that tablets containing caffeine had high solubility in acidic solutions, and in this study, caffeine was expected to dissolve quickly in simulated gastric fluid. The rapid dissolution of materials within the tablet matrix (e.g., caffeine) increases tablet porosity and induces rapid breakup [73], leading to increased stress concentration, cracking and fragmentation under internal pressure.

##### Type 2 Tablets (MCC–Caffeine Tablet)

MCC is generally used as a filler and binder because it can provide mechanical strength and compressibility [74]. However, MCC may resist swelling [32] and increase internal stress during the loss of caffeine via mass transfer, in line with the findings of Abdullah et al. [75], who reported that MCC can act as a rigid matrix, allowing caffeine dissolution to generate concentrated stresses in the matrix (MCC and caffeine). When MCC is present in acidic conditions (SGF), it has been shown to resist degradation [56,57], and the mechanical strength can even increase, as observed in Type 2 tablets after 3 min in SGF. Therefore, the acid penetration resistance and rigidity of MCC can generate stress gradients across the tablet, especially if the outer layers dissolve faster than the core. The MCC promotes tablet breakup (Type 2) by absorbing water, leading to swelling and mechanical rupture [35]. The combined effects include a potential imbalance between swelling and dissolution, in which MCC may swell slightly before collapsing, while caffeine dissolves, creating uneven stress distributions. As pores form and liquid penetrates, capillary pressure can exert additional tensile stresses. Tensile stresses may be created from swelling [76] and void formation, shear stresses may occur at the MCC–caffeine interfaces, and compressive stresses may happen if swelling occurs before hydrolysis dominates.

##### Type 3 Tablets (Pectin–Caffeine Tablet)

In tablets containing pectin and caffeine (Type 3), gelation of pectin occurs in environments containing calcium ions or acidic conditions, particularly for low-methoxyl pectin [30]. When pectin absorbs water, it experiences ionic crosslinking that generates swelling pressure and leads to expansion, which exerts internal stress (axial and radial stress) within the tablets [31]. Interfacial shear stress may occur, since caffeine does not swell, and differential swelling creates shear stresses at its interface [76], which can affect drug release.

#### 3.5.2. Approximate Calculation of Interfacial Shear Stress and Maximum Radial Shear Stress Within Tablets

Klinzing et al. [76] showed that differential shrinkage/swelling within a tablet matrix generates significant internal stresses that influence structural integrity and drug release. Lame theory has been applied to analyse the stress in cylinder dies during tablet compaction [77]. In this study, we have modelled the tablet as an axisymmetric bilayer cylinder elastic solid with constant, isotropic swelling strains and without plastic deformation or viscoelastic time dependence. Therefore, this work used Lame theory to calculate the interfacial shear stress between layers.

The misfit strain was defined by the following equation:

(2) $$∆\varepsilon =\varepsilon_{1}-\varepsilon_{2}$$

where Δ*ε* refers to the misfit strain, *ε*_1_ and *ε*_2_ refer to the swelling strain of layers 1 and 2 (middle), respectively.

The interfacial shear stress was defined by the following equation:

(3) $$\tau_{interface}\approx G_{eff}\times ∆\epsilon$$

where *τ_interface_* refers to the interfacial shear stress, *G_eff_* refers to the modulus of rigidity.

The modulus of rigidity was defined by the following equation [78]:

(4) $$G_{eff}=\frac{E_{eff}}{2(1+V_{eff})}$$

where *E_eff_* refers to the effective Young’s modulus from 1 min measurements for different tablet types in different solutions, and *V*_eff_ is Poisson’s ratio, which is assumed to be 0.3 for most materials, such as MCC [63,79].

The swelling strain for MCC is about 2–3% [76]. A change in the moisture content can affect the viscoelastic behaviour in hydrated matrices [80], and Mahiuddin et al. [80] found that an acidic environment reduced the water uptake rate for tablets under aqueous conditions. Therefore, a swelling strain of 3% was assumed for Type 2 tablets in SGF, while for Types 1 and 2 tablets in water, the swelling strain was assumed to be 2%. Pectin experiences swelling because of gelation. It has been reported that low-solid pectin hydrogel at 30 °C experiences a range of swelling strains between 10 and 12% [81]. The swelling strain increases with increasing temperature [81], and a swelling strain of 10% was assumed at the lower end of the 10–12% range since the temperature in these experiments was 30 °C. The calculation results for the interfacial shear stresses are illustrated in Table 3.

The step-by-step derivation is illustrated in Appendix A.3.

The boundary conditions require stress continuity across the interfaces, displacement continuity on both sides, and zero stress at the free surface. The maximum radial stress may be obtained from the following equation:

(5) $$\sigma_{r,max}\approx \frac{E_{1}h_{1}(\varepsilon_{1}-\epsilon_{2})}{\left(h_{1}+h_{2})(1-V_{eff}\right)}$$

where *E*_1_ refers to Young’s modulus, *h*_1_ and *h*_2_ refer to the thickness of the first layer and second layer, assumed equal (*h*_1_* = h*_2_). The formula is based on the assumption that the stress is concentrated at the MCC–caffeine interface. The calculation results of the maximum radial shear stress are illustrated in Table 3.

The interfacial shear stress of the Type 1 tablet is predicted to be low (0.003 ± 0.0007) MPa, and the radial shear stress is also low (0.007 ± 0.002) MPa (Table 3). These values suggest weak bonding and minimal internal stress to maintain structural integrity, which corresponds to the rapid breakup in water. The interfacial shear stress of the Type 2 tablet in water is predicted to be (0.007 ± 0.0009) MPa, which is about two times larger than the maximum radial shear stress value of Type 1 tablets (Table 3). It supported the observation that, in Type 2 tablets, the caffeine dissolves, increasing the stress gradients to lead to tablet breakup. The interfacial shear stress for Type 2 tablets in SGF ((0.005 ± 0.0007) MPa) was lower than that of Type 2 tablets in water, which means that the MCC with caffeine in SGF undergoes a similar disintegration process. The maximum radial shear stress of the Type 2 tablet in water ((0.02 ± 0.002) MPa) is predicted to be two times larger than that for the tablet in SGF, which suggests that the internal pressure for the Type 2 tablet in SGF will have smaller pressure gradients to lead to a more stabilised structure as the internal pressure develops under both conditions (Table 3). The Type 3 tablets showed high interfacial stress, predicted to be (0.03 ± 0.004) MPa, and large radial shear stresses, predicted to be (0.07 ± 0.01) MPa, compared with the other types of tablets, which reflected the swelling pressure related to the pectin expansion during gelation (Table 3).

The sample calculation is illustrated in Appendix A.4, with the estimated fracture strength shown in Table A3. The plausible fracture strength was estimated as a theoretical reference value to help assess the localised stress concentrations within the tablet matrix because the calculated interfacial shear stresses (0.003–0.03 MPa) are still orders of magnitude lower than the tensile/shear strength of the material. The maximum radial shear stress was calculated to range from 0.007 to 0.02 MPa, which is close to the lower fracture strength (0.02 ± 0.004 MPa). Young’s moduli decrease at greater moisture contents [82]. Equation (A8) in Appendix A.4 shows that the fracture strength of the porous tablet (σ*) decreases as the Young’s moduli are reduced, so lower Young’s moduli at higher moisture contents are likely to decrease the fracture strength below the calculated range values (0.02–0.05 MPa). The maximum radial stress for the Type 1 and 2 tablets ranged from 0.007 MPa to 0.02 MPa. The consideration further suggests that the true fracture strength is within the range of maximum radial shear stresses for the wet, porous matrix. Therefore, the significant radial shear stress may be the reason for the breakup that occurred for all tablet types.

## 4. Conclusions

A new beaker system was developed to study the breakup process during digestion. Three special types of tablets were tested. Types 1 and 2 tablets both contained microcrystalline cellulose but differed in their preparation methods: Type 1 tablets were produced via physical mixing, while Type 2 tablets were prepared using spray drying. Type 2 tablets showed a consistent breakup time of (1.5 ± 0.2) minutes in water and (1.8 ± 0.04) minutes in simulated gastric fluid (SGF). Type 3 tablets, consisting of pectin and caffeine, showed a pure dissolution process in the water system. These tablets showed initial values of Young’s moduli that are close to those of foods.

The Young’s moduli were measured at different digestion time points, which helped to identify the interaction between texture and breakup. Types 1 and 2 tablets experienced a drop in Young’s modulus, which corresponded to the disintegration that occurred. Type 3 tablets showed a generally increasing modulus trend after two minutes, corresponding to the gelation process for pectin. Fracturability trends further supported these observations. SGF gave more stable mechanical behaviour compared with water due to the acid resistance of the MCC and the slow water uptake behaviour in SGF, which caused slow swelling, resisting the breakup phenomenon. Micro-CT tests showed that the Type 2 tablet in the water system had more cracks than the tablet in SGF, which corresponded to the Type 2 tablet breaking up more easily in water. Estimates for the maximum radial and interfacial shear stresses based on Lame theory support these observations and emphasise the importance of swelling, caffeine dissolution, and pectin gelation as vital processes in food disintegration. A limitation of this study is that the real food matrix is more complex than the developed tablet-based model, but the current study provides some fundamental insight into the interaction between food texture and breakup behaviour. This mechanistic understanding can help control nutrient release to improve food sustainability through the design of food structures.

## Figures and Tables

**Table 1:** The compositions of the different tablets created in this study.

Tablet Type	Formulation Components	Composition (*w*/*w* %)	Tablet Mass (g)
Type 1 tablet	MCC + caffeine	90% MCC + 10% caffeine	0.18 g MCC + 0.02 g caffeine
Type 2 tablet	Spray-dried powder (MCC + caffeine)	90% MCC + 10% caffeine	0.2 g
Type 3 tablet	Spray-dried powder (pectin + caffeine)	90% pectin + 10% caffeine	0.2 g

**Table 2:** Comparison of the values of Young’s moduli for existing food materials.

Materials	Reported Young’s Modulus (MPa)	Conditions	Reference
Apple	1.84–3.65	Different test speeds	[29]
Banana	0.028–0.076	Different test speeds	[29]
Peaches	1.03	/	[38,39]
Pear	5.3	/	[38,39]
	1.42–2.71	Different test speeds	[29]
Soybean	125–126	moisture content of 13%	[39,40]
Goli red bean	93.06–253.26	Varying moisture content	[39]
Akhtar red bean	82.28–224.94	Varying moisture content	[39]
Japanese radish	Different boiling times 0.3–1.8	Different boiling times	[41]
Cheese	Different moisture contents 0.13–0.24	Different moisture contents	[42]
Carrot	Different boiling times 0.5–3	Different boiling times	[41]
	Different test speeds 6.1–8.8	Different test speeds	[29]

**Table 3:** Comparison of estimated interfacial and maximum radial shear stresses for different types of tablets in water and simulated gastric fluid (SGF).

Different Types of Tablets	Different Solutions	Interfacial Shear Stress (MPa)
Type 1 tablet	water	0.003 ± 0.0007
Type 2 tablet	water	0.007 ± 0.0009
	SGF	0.005 ± 0.0007
Type 3 tablets	water	0.03 ± 0.004

**Figure A1:**
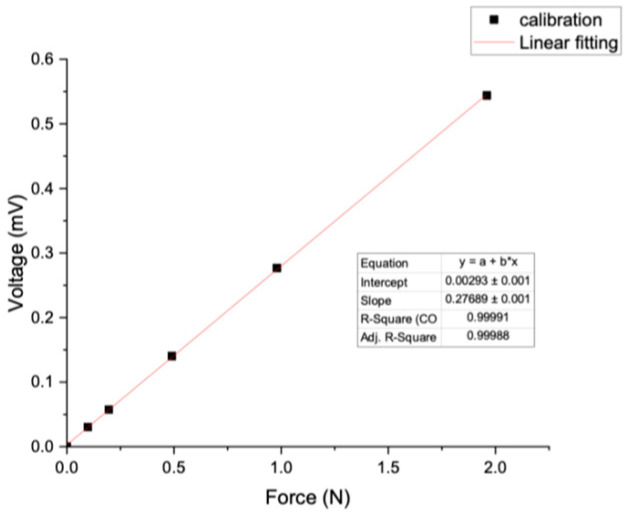
The calibration curve of the relationship between force and voltage.

## Data Availability

The original contributions presented in this study are included in the article. Further inquiries can be directed to the corresponding author.

## Appendix A

### Appendix A.1. Force–Time Curve

The force–time curve was calculated from the raw voltage–time data recorded during the tablet texture tests for calculating Young’s modulus. The linear calibration between force and voltage was tested in three experiments, as shown in Figure A1. The calibration procedure used the standard weights on the load cell and recorded the corresponding voltage output (mV). The relationship between voltage and force, which is defined by the following equation:

(A1) F=aV+b

where *F* refers to the force, which is calculated from mass (g) × gravitational acceleration (g = 9.8 m/s^2^), and *V* refers to the voltage from the load cell data collection. Figure A1 shows the calibration results, where the slope is 0.28 for converting the voltage (mV) to force (N).

As shown in Figure A1, the slope of the equation for the relationship between force and voltage is 0.28 N/mV, which was used to convert the voltage signal to force values. The sample calculation gives $Force=\;\frac{Voltage}{Slope\;value}=\frac{0.12\;mV}{0.28\;N/mV}=0.43\;N$. A representative force–time curve is shown in Figure A2.

### Appendix A.2. ANOVA Analysis

The analysis of variance (ANOVA) table results for the Young’s moduli for different types of tablets at different time points and in different solutions (water or simulated gastric fluid (SGF)), 95% confidence level, show significant differences for time points, tablet types, solutions, and the interaction between the time point and tablet types and the interaction between time points and solution difference. The Young’s modulus was analysed using a two-way ANOVA between different time points and different types of tablets, as shown in Table A1.

**Table A1:** ANOVA for the Young’s modulus across time points for each tablet type in water.

Source of Variation	Degree of Freedom (df)	Sum of Squares (SS)	Mean Square (MS)	*p*-Value
Time points	6	0.8	0.1	2 × 10^−5^
Tablet types	2	1.3	0.7	5 × 10^−10^
Interaction: time points × Tablet types	12	3.1	0.3	3 × 10^−11^

The two-way ANOVA with replication was used to analyse the effects of different time points and tablet types on Young’s modulus. The *p*-value for the time points factor is 2 × 10^−5^ lower than 0.05, indicating a significant difference between the time points and Young’s modulus (Table A1). The significant difference can also be found in tablet types, which is supported by the *p*-value (5 × 10^−10^) lower than 0.05 (Table A1). The interaction between time points and tablet types was significant in Table A1, suggesting that the effect of time on Young’s modulus depended on tablet types.

A two-way ANOVA was also applied to analyse the interaction between time points within Type 2 tablets and different solutions, as shown in Table A2, as a representative analysis.

**Table A2:** ANOVA for the Young’s modulus across time points for the Type 2 tablet type in different solutions (water and simulated gastric fluid).

Source of Variation	Degree of Freedom (df)	Sum of Squares (SS)	Mean Square (MS)	*p*-Value
Time points	4	0.098	0.02	0.1
Solutions	1	0.8	0.8	4 × 10^−8^
Interaction: time points × solutions	4	0.07	0.02	0.2

The significantly large effect of solutions is shown in a two-way ANOVA, with a *p*-value of 4 × 10^−8^. The *p*-value for the interaction between the time points and solutions shows 0.2, while the time points factor is 0.1 (Table A2). These results indicated that various times and time × solution interactions do not have significant effects for this comparison between solutions.

### Appendix A.3. Radial Shear Stress Calculation

The radial stress using Lame theory is defined by the following equation [83]:

(A2) $$\sigma_{r}^{\left(i\right)}=A_{i}-\frac{B_{i}}{r^{2}}$$

where $\sigma_{r}^{\left(i\right)}$ refers to the radial stress, *A_i_* and *B_i_* refer to Lame constants, and *r* refers to the difference in radius between the two layers.

The hoop stress is defined by the following equation [83]:

(A3) $$\sigma_{\theta }^{\left(i\right)}=A_{i}+\frac{B_{i}}{r^{2}}$$

where $\sigma_{\theta }^{\left(i\right)}$ refers to the hoop stress.

The radial displacement, as each layer has a shrinkage strain, is defined by the following equation:

(A4) $$u^{\left(i\right)}\left(r\right)=\left(\frac{1+V_{eff}}{E}\right)\times \left(A_{i}r-\frac{B_{i}}{r}\right)+\epsilon_{i}r$$

where $u^{\left(i\right)}\left(r\right)$ refers to the radial displacement, and *ε_I_* refers to the swelling strain for the layer. The detailed derivation step can help to get the final Equation (5) in the paper.

### Appendix A.4. Plausible Fracture Strength Calculation

The relationship between the Young’s modulus and relative density is defined by the following equation [84]:

(A5) $$\frac{E^{*}}{E_{s}}={\left(\frac{\rho^{*}}{\rho_{s}}\right)}^{2}$$

where *ρ**/*ρ_s_* refers to the relative density of materials. *E** refers to the effective Young’s modulus of the porous tablet, and *E_s_* refers to the Young’s modulus of the cell-wall solid (pectin or MCC).

The relationship between the fracture strength and relative density is defined by the following equation [84]:

(A6) $$\frac{\sigma^{*}}{\sigma_{fs}}={0.2\left(\frac{\rho^{*}}{\rho_{s}}\right)}^{\frac{3}{2}}$$

where *σ** refers to the fracture strength of the porous tablet, and *σ_fs_* refers to the fracture strength of the cell-wall solid (pectin or MCC).

Eliminating (*ρ**/*ρ_s_*) between the two relations is defined by the following equation:

(A7) $$\frac{\sigma^{*}}{\sigma_{fs}}={0.2\left(\frac{E^{*}}{E_{s}}\right)}^{\frac{3}{4}}$$

The Young’s modulus and fracture strength of the cell-wall solid are constant, as the same material was used for each tablet type. The wet tablet was modelled as an open-cell polymer network. $\frac{\sigma_{fs}}{E_{s}}$ can be taken as 0.03, which is a typical value for isotropic plastic foams that may represent many polymers [84]. Then, using $\frac{\sigma_{fs}}{E_{s}}$, Equation (3) may be rearranged as follows for the fracture strength:

(A8) $$\sigma^{*}=0.2\times \frac{\sigma_{fs}}{E_{s}}\times {\left(E_{s}\right)}^{\frac{1}{4}}\times {\left(E^{*}\right)}^{\frac{3}{4}}$$

Types 1 and 2 tablets: The Young’s modulus value for MCC, which is considered a cell-wall solid material, is within the range of 4–9 GPa, within the moisture content range of 0–10% [82]. Therefore, a sample calculation for fracture strength is as follows: $\sigma^{*}=0.2\times 0.03\times {\left(4\times 1000\right)}^{\frac{1}{4}}\times {\left(0.3\right)}^{\frac{3}{4}}=0.02\;MPa$. The fracture strength is from 0.02 MPa to 0.05 MPa for Type 1 and 2 tablets, as shown in Table 1.

In the experiments here, Type 3 tablets gelled very quickly, which made them plastic from the beginning of the dissolution process. This plasticity made them very resistant to the fracture process, so the above calculation has little physical meaning for the Type 3 tablets, and the above fracture calculations for Types 1 and 2 tablets have not been repeated for Type 3 tablets. The gelling of the Type 3 tablets has been shown in Figure 5c.

**Table A3:** Estimated fracture strengths for different tablets in different solutions.

Different Types of Tablets	Different Solutions	Upper Fracture Strength (MPa)	Lower Fracture Strength (MPa)	Maximum Radial Shear Stress (MPa)
Type 1 tablets	water	0.03 ± 0.005	0.02 ± 0.004	0.007 ± 0.002
Type 2 tablets	water	0.05 ± 0.005	0.04 ± 0.004	0.02 ± 0.002
	SGF	0.03 ± 0.003	0.03 ± 0.003	0.01 ± 0.002
